# Determinants of Bird Species Literacy—Activity/Interest and Specialization Are More Important Than Socio-Demographic Variables

**DOI:** 10.3390/ani11061595

**Published:** 2021-05-28

**Authors:** Christoph Randler, Felicitas Heil

**Affiliations:** Didaktik der Biologie, Fachbereich Biologie, Eberhard Karls Universität Tübingen, D-72076 Tübingen, Germany; felicitas.heil@student.uni-tuebingen.de

**Keywords:** age, birding specialization, bird species knowledge, garden owners, urban–rural dichotomy, distance to next forest patch, gender

## Abstract

**Simple Summary:**

Biodiversity is declining around the world and knowledge about biodiversity declines in a similar way. In this study, we addressed predictors of species knowledge, i.e., the question of what influences or causes species knowledge. The focus was knowledge about common bird species in Germany. Data were collected from July to October 2020 via an online questionnaire, containing demographic data, engagement in birdwatching, interest/activity, and images of 28 bird species native to Germany. Data were collected from adult students, lecturers and administrative staff of the Eberhard Karls University Tübingen. Men identified more species than women, garden owners more than non-owners. Hometown size had no influence. Employees scored higher than students. However, we found that birding specialization was the most important predictor, followed by bird-related interest/activity. We suggest including such questions in addition to conventional demographic questions in the future.

**Abstract:**

Biodiversity is declining, and knowledge about biodiversity declines in a similar way. Previous studies have already addressed predictors of species knowledge. Here, we studied bird species knowledge related to demographics, but also to individual differences in affinity to nature, by including (i) birding specialization and (ii) bird-related activities/interest. Data were collected from July to October 2020 via an online questionnaire, containing demographic data, birding specialization, interest/activity, and images of 28 bird species native to Germany. Participants were adult students, lecturers and administrative staff of the Eberhard Karls University Tübingen. A total of 1967 questionnaires were returned in this study (35.3% male, 53.8% students, 69% had access to a garden). Mean identification score was 16.31 ± 6.38. Thus, participants were able to identify more than half of the species (total species *n* = 28). Men identified more species than women, garden owners had higher identification scores than non-owners, while hometown size was not significant. A distance to the next forest patch >10 km was related to lower identification scores. Employees scored higher than students. Correlation between species knowledge and birding specialization was high, as was the correlation with bird interest/activity. Higher scores were found in older people. In the linear univariate model, birding specialization and bird interest were the most influential predictors of species knowledge, followed by distance to next forest patch and occupation (student vs employees). Other variables were not significant. We suggest including such measures (interest, attitude, etc.) into further studies and move forward from the urban–rural narrative to more complex analyses of living circumstances.

## 1. Introduction

Biodiversity is declining around the globe, and knowledge about biodiversity continues to decline in a similar manner [1,2]. As a result, biodiversity knowledge is becoming an increasingly important issue and it is recognized worldwide as an important environmental task [3,4]. At the United Nations Conference on Environment and Development in Rio de Janeiro (1992), a convention on biological diversity was adopted by 179 countries, with the focus on biodiversity protection and to uphold the loss of biodiversity knowledge through teaching and learning.

However, a uniform definition of biological diversity is not easy, and it is often technically defined [5]. Biodiversity itself is made up of individual species, but biodiversity is more than species diversity and includes genetic and ecosystem diversity. Knowledge of plant and animal species as well as the identification of individual species is essential for understanding ecology and for conservation efforts [3,6]. In addition, the success of various nature conservation measures depends on the population’s perception of biological diversity and the perception of species [4,7]. Dallimer [8] et al. (2012), for example, reported that people were not accurately able to assess the species diversity in a landscape, but as an individual’s identification skills improved, so did their ability to accurately gauge levels of biodiversity [8]. Interestingly, “appealing” bird species have better conservation status [7], thus increasing species knowledge is an important aspect of teaching and learning [3]. Enthusiasm for biodiversity can be increased with the help of the concept of species knowledge [1]. Concerning specific taxa, birds are among the most charismatic taxonomic group [9]. Therefore, basic knowledge of common bird species and how to identify different bird species is recognized as an essential part of understanding the framework of ecosystems [10,11]. Identifying the determinants of species knowledge is an important aspect to target educational needs [12]. The influence of socio-demographic factors for species knowledge has been addressed in previous studies in adults and adolescents, mainly based on the demographic variables age, gender, and habitation (such as the urban versus rural dichotomy; [13]).

Age in particular has been recorded by various studies as determinant. However, age effects have never been studied in a comprehensive manner from childhood to seniors. Therefore, the evidence is somewhat contradictory but can be explained by looking at the different studies and age groups. In 4–12-year-old children, species identification skills increased with increasing age [14], but only up to the age of eight or nine years, after which species knowledge seemed to decrease again. However, in adolescence, Randler [3] showed that species knowledge continues to increase up to the age of 14, but then tends to level off again. These two results might not be contradictory but might be owed to different sampling techniques and studied cohorts, e.g., in different countries and different school systems. However, in university students, skills improved from student teachers to university teachers (Kaasinen, 2009 cited [6]). Concerning adults from the general public, nearly all studies showed an increasing species identification knowledge parallel to an increasing age [1,15,16,17,18], which might result from free-choice, informal learning, meaning that older participants had spent more time learning about animal species, either unintentionally or by incidental or informal learning [15].

Concerning gender, differences in identification scores remain equivocal. In the kindergarten age, Randler and Wieland [19] found no difference between boys and girls in species knowledge. In a study by Hummel et al. [9] based on 852 schoolchildren (average age 11.48 years) from different countries (Colombia, Germany, Slovakia, and Turkey) female participants showed a greater interest in ornithology, but there was no difference in the cognitive domain. Other studies in adolescents found that boys in the UK had significantly greater wildlife knowledge than girls [14]. Similarly, Brazilian boys scored better in snake identification than girls [20]. In contrast, girls scored higher than boys in vertebrate species knowledge in secondary school students in Germany [2]. Concerning adults, Mmassy and Røsekaft [18] examined the knowledge of bird species in 330 participants living around the Serengeti. Here, men scored significantly better than women. Hooykaas and colleagues [1] found higher species literacy in men in the general Dutch public, while there was no gender difference in adult Israelis, also drawn from a general public population [17]. Women scored higher than men, however, in some other studies [21,22]. Different approaches have been invoked to explain gender patterns, ranging from evolutionary psychology (e.g., men were more involved in hunting) to educational psychology, e.g., girls perform better in biology subjects at school [23].

Living situation, and especially the urban–rural dichotomy was also assessed as an explanatory variable in species knowledge mainly based on the hypothesis that rural people are more connected to nature and may experience nature directly more often or intensely. Most studies confirmed this hypothesis. For example, urban teacher students performed worse than rural ones [6]. In the Israelian public, lower species knowledge was found in urban dwellers [17]. Similarly, in Brazil rural students performed better at identifying snakes than urbanites [20], and in China the same pattern was observed concerning frogs [22]. However, in Puerto Rico, people living in rural communities were found to be less knowledgeable about birds than urban residents [24]. Mmassy and Røskaft [18] investigated the ability to recognize bird species based on images with respect to the living situation. It was found that people who live closer to the Serengeti National Park have a poorer ability to correctly identify bird species than people who live further away [18].

Therefore, the living situation seems another important predictor. Hooykaas and colleagues [1] found owning a garden to be a statistically significant predictor of species knowledge. Other local effects, such as distance to nature may also influence the species knowledge [17]. For example, park visitors showed a significantly higher species knowledge compared to non-visitors within the same city [15].

In addition to socio-demographic factors, individual differences in activity and interest may have an important impact on species knowledge. Animal-related activities, such as visiting zoos, going into nature to observe animals, but also reading books or on the internet about animals, were correlated with species knowledge [16]. Interest in nature correlated positively with higher species identification scores [6] and the interest in identifying animal species was correlated with the number of correctly identified species in teacher students [25]. Additionally, attitudes toward nature and animals were significantly related to species knowledge [1]. Participation in animal-related activities showed a strong association with interest in animal species [9]. Palmberg et al. [6] and Cox and Gaston [26] similarly observed a positive correlation in their respective studies. These studies point at the importance of differences in animal-related activities, attitudes, and interest as predictors of species knowledge.

A topic previously unaddressed in species knowledge is recreation specialization [27]. Recreation specialization usually considers three dimensions: first, skill and knowledge in a given activity, in this case, number of bird species one can identify by sound and sight. Second, behavior, which is usually measured by time and effort invested in the activity, and, finally, psychological and behavioral commitment [28]. Especially in birdwatching, there is a bulk of literature on birding specialization, which is a conceptual framework that addresses the aspect of recreation. Birders can be sorted along a gradient from very beginners and only casual observers to highly specialized birders that spend a considerable amount of time and money for their recreation activity [28].

### The Present Study

The research question was to assess determinants of bird species knowledge and the relationship with two important predictors: (i) birding specialization and (ii) bird related activities/interest by controlling for well-known demographic variables. Some studies have addressed these predictors above, mainly based on demographics. Only few tried to address the differences in affinity to nature. While most approaches were concerned in comparing men/women or the urban–rural dichotomy (or sometimes gradients), we here add to previous work by including variables such as birding specialization and bird-related activities as individual aspects. We address these differences by basing the study on validated questionnaires. The sample of university students and employees of the Eberhard Karls University Tuebingen was chosen because we wanted to test the new measurements in relation to bird species knowledge, and we assumed that the relationship between birding specialization and bird species knowledge, as well as the relationship between bird interest/activity and bird species knowledge should be a universal result, and therefore, a representative sample was not required.

Hypotheses of the present study were:

1. Bird species knowledge and birding specialization are correlated.
2. Bird species knowledge and bird commitment are correlated.
3. Men score higher than women in bird species knowledge.
4. Distance to the next forest patch is related to bird species knowledge.
5. Garden owners score higher than non-owners on bird species knowledge.
6. Hometown size is related to bird species knowledge with higher scores in rural areas.

## 2. Material and Methods

### 2.1. Participants and Data Collection

The questionnaire was sent to all university students and staff (lecturers, administrative staff) of the Eberhard Karls University of Tübingen via an institutional round mail. Tuebingen host about 27,000 students, 2200 administrative staff, and 4900 teaching staff and scientists. The university has a small botanical garden (10 ha). Data were collected from July 2020 to October 2020. For this purpose, a questionnaire with 28 bird species native to Germany was made available via the online questionnaire program SoSciSurvey (data downloaded on 14 October 2020). The study is based on a bachelor thesis (F.H.) and a formal ethical approval is not required (see Institutional Review Board Statement below).

### 2.2. Questionnaire

#### 2.2.1. Demographic Data

The first part of the questionnaire contained demographic data: age, gender, occupation (coded into student or employee). Hometown size was coded into the following categories: <2000/2000–5000/5000–20,000/20,000–100,000/>100,000. These categories were based on [29]. Distance to the next forest patch was categorized into <1 km/1 to 5 km/6 to 10 km/>10 km. Garden ownership was coded into yes or no. We obtained 2139 questionnaires. Among those 172 did not provide answers on the bird identification questions. Thus, 1967 questionnaires remained valid for further 183 analysis. A total of 695 participants were male (35.3%), 1218 female, 15 diverse, and 39 preferred not to answer the question concerning gender. Of those questioned, 1047 were students (53.8%), 898 were employees, and 22 did not respond to this question. A. total of 1353 participants possessed a garden, 610 did not (4 without answer). Distance to the next forest patch was less than 1 km in 1040 respondents, between 1 and 5 km in 828, 6 to 10 km in 78 participants, more than 10 km in 19, and 2 persons did not answer. Table 1 depicts the sample characteristics split by gender.

#### 2.2.2. Birding Specialization

Birding specialization was assessed with five questions (following [28]): Number of bird species to be able to identify by sight (category up to 10/up to 20/up to 50/up to 100/more than 100 and by sound (which means song or call; category up to 5/up to 10/up to 20/up to 50/more than 50). Self-assessment of one’s own ornithological expertise, ranging from 1 (novice) to 5 (expert). These three questions formed the skill/competence component. The behavior component included the number of bird excursions during the last year (none/1–2/up to 5/up to 10/more than 10), and the number of bird books at home (none/1–2/up to 5/up to 10/>10). Cronbach’s alpha was 0.826 concerning the five questions. The sum of all five items was calculated and used as a score of birding specialization. Additionally and separately, we calculated the subscale of skill/competence in the self-assessment of bird species knowledge, containing only the three questions about number of species being able to identify by sound, appearance and the self-assessment of knowledge.

#### 2.2.3. Bird Related Activities and Interest

Bird-related activities and interest were measured with items provided by Randler [16] and Hummel et al. ([9]; see Table 1). The items were Likert-scaled from 1 to 5. An exploratory factor analysis (EFA) with principal component extraction and varimax rotation was applied on the interest/bird-related activity scale since the items have been taken from different previous studies. Residuals from the regression (EFA) were saved as factor scores for further processing. This method saves the factor scores and weighs the relative importance of each item to the scale.

#### 2.2.4. Bird Identification Scores

The identification part of the questionnaire covered the knowledge of species and consisted of photographs showing 28 bird species native to Germany. In the study period July to September, these bird species were present in Germany. All images were colored, as color is an important identification feature in birds. In addition, we presented the bird images one after another to avoid distraction. The participants were asked to identify the respective bird species in a text box. The list of bird species were taken from previous work [3]. The species were chosen because of their abundance and occurrence in Germany. All bird species are representative in terms of bird order/families (taxonomic) and according to their number of breeding pairs, their abundance and visibility ([3]; see Appendix A). We covered most of the orders of breeding species in Germany (at least one representative of the order), and focused on the distribution, i.e., that the bird species were distributed widespread across the country. Therefore, procellariforms or alcids, for example, were not used. Further, we checked the abundance with the breeding bird data [30] and included 10 of the most 20 common bird species. However, to avoid bias due to the high number of species within the passerines, not all of the 20 most common bird species were included. Thus, the selected sample represents. a balance between covering the most bird orders and the most common species. For the most part, pictures of the bird species were photographed by the authors (CR) and were freely available for the questionnaire. The images of the tawny owl (*Strix aluco*) and blackcap (*Sylvia atricapilla*) were taken from the website Pixabay (license-free) [31,32].

### 2.3. Coding of Correct Answers

Coding of the answers followed a partial credit model [2,3]. If a bird species was identified correctly, the value 1 was assigned (e.g., Tufted Duck *Aythya fuligula*). Foreign languages and spelling errors were allowed. If the correct family was mentioned, for example the blackbird, the family of thrushes (Turdidae), the subfamily, tribe or genus, or a bird species of the same family, the value 0.5 was assigned as a partial credit to test the species knowledge in general. The value 0 was assigned as soon as the indication of the participants could not meet any of the criteria. If no response was entered into the text box, the value −99 was specified as missing data, but was later coded into 0. Exceptions and other rules were: Question marks were not counted; text in brackets was not counted; synonyms and everyday language were allowed; trivializations were allowed.

### 2.4. Statistical Analysis

Our analytical strategy followed a two-tier procedure. As previous studies were mainly based on demographics, we made first an analysis with bivariate statistics to make the results comparable to previous work. In the second step, we used a complex general linear model including the differences in activity/interest and birding specialization. Cronbach’s alpha was used to measure internal consistency of the birding specialization scale as it is an existing scale/measurement. Mann–Whitney-U tests were used to compare binary variables (gender, occupation), Kruskal–Wallis tests were used when comparing more than two groups. Pearson’s correlations were used for analyzing relationships. We used a univariate general linear model with the predictors age, gender, occupation, distance to next forest patch, owning a garden, hometown size, birding specialization, and interest/activities. The full model containing all these variables was inspected and subsequently, the variables with the highest *p*-values were deleted in a stepwise procedure until only significant predictors remained in the model. This was labeled the final model. SPSS 26 was used for analyses. As we did more than one test on the same dataset, Bonferroni correction should have been used. However, most of the results are significant on the ≤0.001 level. Ten comparisons on the 0.05 level would lead to a Bonferroni corrected α level of 0.005.

## 3. Results

Mean identification score was 16.31 ± 6.38 (mean ± SD). Thus, participants were able to identify more than half of the species (total species *n* = 28). An EFA was applied on the items concerning bird related activities/interest, as this scale has not been established previously. The EFA extracted one factor with an Eigenvalue of 3.35 (55.78% of variance explained; see Table 2). Factor loadings and items are presented in Table 3.

**Figure 1:**
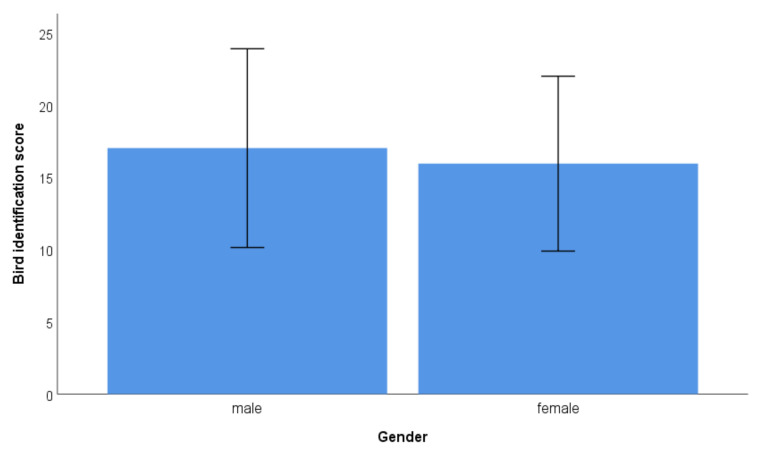
Bird identification scores in relation to gender (mean and SD given).

Men were able to identify more species than women (mean ± SD: 17.0 ± 6.9 versus 15.9 ± 6.0; Figure 1). This difference was significant (Mann–Whitney-U test: Z = 3.423, df = 1911, *p* = 0.001).

**Figure 2:**
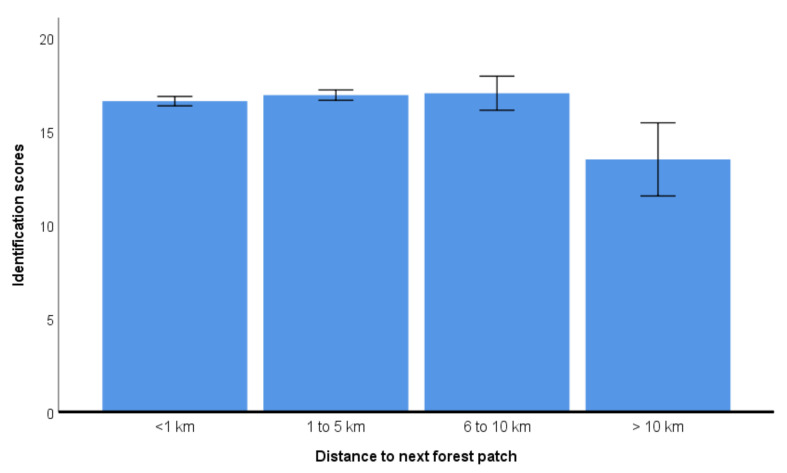
Bird identification scores in relation to distance to next forest patch (mean and 95 CI given derived from the general linear model).

Employees scored higher in identification than students (17.2 ± 6.3 versus 15.5 ± 6.3). This difference was significant (Mann–Whitney-U test: *Z* = −5.902, df = 1943, *p* < 0.001). Garden owners also showed higher identification scores than non-owners (16.6 ± 6.4 versus 15.7 ± 6.4; Mann–Whitney-U test: Z = −3.124, df = 1961, *p* = 0.002). Hometown size was not significantly different between the groups (Kruskal–Wallis test: *H* = 0.697, *p* = 0.952), while distance to the next forest patch showed a significant influence (Kruskal–Wallis Test: *H* = 19.53, *p* < 0.001; Figure 2). Correlation between species knowledge and the self-assessment of the birding specialization subscale was high (*r* = 0.729, *p* < 0.001, *n* = 1888), as was the correlation with the full birding specialization scale (*r* = 0.731, *p* < 0.001, *n* = 1888). The identification scores also correlated significantly with the interest/activity scale (*r* = 0.592, *p* < 0.001, *n* = 1888). Age was correlated with identification scores, showing higher scores in older people (*r* = 0.171, *p* < 0.001). In the final general linear model, all factors were assessed simultaneously, and birding specialization and bird interest were the most influential predictors of species knowledge considering their partial eta^2^ (Table 4). Both predictors interest/activity and birding specialization were statistical predictors without collinearity (VIF = 1.6).

Concerning occupation, employees performed better than students. With respect to distance to the next forest patch, people living farther than 10 km away from the next forest patch scored significantly lower. Post-hoc analyses revealed differences between the category >10 km and all other categories (Figure 2).

## 4. Discussion

Here, we addressed additional explanatory variables for bird species knowledge, such as activity/interest and birding specialization. This study corroborated previous work but reported new aspects on the influential factors of bird species knowledge. Further, our results differed whether we used bivariate or multivariate analyses. The hypotheses H1, H2, H4 were confirmed; H3 and H5 in parts, while H6 could not be confirmed. 

Concerning gender, men scored consistently higher than women, which confirms previous work on this aspect. Usually this is explained with evolutionary history, such as that men were hunters [23]. However, more recent studies [2] showed higher scores in girls (but see also [3]). As those studies have been conducted in Germany in secondary school students, this might be a Germany-specific result. However, this present study also collected data from German students and working adults, thus, it might be school specific. In most school subjects in Germany, girls outperform boys, and in biology, girls are also more motivated and interested. As the gender differences faded in the GLM, we hypothesize that the differing results in gender differences are not simply a gender effect but depend primarily on other aspects/variables or cohorts assessed in the different previous studies. However, another study with a reasonable sample size and complex modelling in the general public [1] reported exactly the expected gender differences (men scored higher than women) despite using control variables. Therefore, future studies examining gender differences should build on assessing more predictor variables and probably meta-analyses may help to address the equivocal results concerning gender. In animal welfare attitudes, the strength of the gender differences is dependent on economic development of the respective countries [23].

Comparable to nearly all studies on adults, age was a significant predictor for species knowledge, but this effect disappeared in the GLM. This correlation is usually explained with lifelong learning; thus, adults learn about species over the course of their lives [1]. However, although this is plausible, this does not exclude a cohort effect, namely that older people received more species training during their school time or lived in a more nature-rich environment during younger years. This is confirmed indeed by a recent study suggesting that differences in species knowledge between age cohorts are also an explanation [2]. Gerl et al. [2] showed a decline of vertebrate species knowledge in school students between 2006 and 2018 indicating a 15% loss in pupils’ species knowledge within the last decade. A change in the curriculum as a reason for this decline in taxonomic knowledge was assumed [2]. Alternatively, a change in biodiversity (especially avian diversity) might also be an explanation, because the depauperating biodiversity may exert an influence on species knowledge. However, this is only a speculation because data are missing to relate species depauperating with knowledge loss. Age may also be related to the working situation because employees scored higher than students in our study. Thus, the variables age and occupation might be somewhat redundant in our study but might be retained in further studies. Probably, interest in birds is related to a given life situation rather than simply age-dependent, e.g., when people start working or may start a family, they might discover their interest in birds (Randler, unpublished data on >2000 birdwatchers). Finally, the age effect may be a compound of life-long learning as suggested by Hooykaas et al. [1] or a cohort effect [2].

Concerning the living situation, garden owners showed a higher identification score compared to non-owners, which fits into previous work [1,26]. However, this result did not prevail in the GLM. Hometown size was also not a significant predictor of species knowledge. This contrasts with some of the studies (e.g., [6]). However, we did not use a simple dichotomy like urban versus rural. Respondents were separated into bins of different village/city sizes depending on the number of inhabitants. This was made in accordance with Gerl et al. [2], who also reported no difference in vertebrate knowledge between urban and rural school students’ [2]. In contrast, in children from eight kindergarten in SW Germany (near Stuttgart), the highest vertebrate knowledge scores were found in a large city and simultaneously in the most rural place [19]. So future studies should take a more detailed look at the living situation apart from the simple urban versus rural narrative. The urban–rural differences found in some previous studies might be related to distance to nature. Distance to the next forest patch might be the better measurement because in more urban areas this distance can be high when living in the center of a city (but beware of exceptions, such as the Central Parc in New York, which is a birding hotspot). Thus, dwellers of large cities usually have a higher distance to nature compared to inhabitants of small villages. This fact may explain the urban–rural dimension. Similarly, garden owners scored higher in previous studies [26], this may also be owed to the fact that gardens are more common and affordable in smaller villages, and these may be located closer to the next nature spot.

Concerning the differences in activity/interest and birding specialization, birding specialization had the largest impact on knowledge scores, followed by interest/activities. This was reported in some previous studies [1,6]. Despite the fact that different studies used different approaches measuring these individual variables, they showed that interest [6] or attitudes [1] were important predictors. We strongly suggest in further studies on species knowledge to include such questions, because it shows that age, gender, and habitation only have a marginal influence on knowledge and species literacy when compared with more complex approaches reflecting individual variables.

Generally, causation cannot be inferred in a cross-sectional design. This means that people with a high species knowledge may prefer to live in a house with garden, or live closer to nature (self-selected), and may spend more time outdoors. This is impossible to test experimentally, but longitudinal studies might give some insight, e.g., when people during life course changes may change habits, and may be studied before and after such changes in a panel study. However, some information comes from the birding initiation study (Randler unpublished), where people reported that moving into a house with garden was one of the triggers of their bird interest.

We also advanced in methodological aspects. Birding specialization is usually measured with self-report, like our study. However, as we have also included a test on bird species knowledge, we are able to show that this self-report measure is strongly correlated with the test results. As far as we are aware, it has never been attempted to test the validity of this self-report measurement. We here show that this self-assessment is a reliable tool for the skill/knowledge scale because it correlated with 0.7 with the bird identification score.

### Limitations

The population for our study were students, teachers, and administrative staff of the Eberhard Karls University Tübingen. Thus, it is not a representative sample from the population in Germany. Workers that did not attend university are missing from the dataset. Nevertheless, we feel that results, especially the relationship between birding specialization and interest in birds with knowledge scores can be generalized to the population. Further, we did not collect data on the highest degree or level of education as another explanatory variable because the university sample is less heterogeneous than an average population sample. Further, the socioeconomic status should be assessed in a representative sample. Similarly, the respondents were much younger than the average German population. These aspects should be considered and added in further studies.

## 5. Conclusions

As a conclusion, we add to previous work on species knowledge by using additional explanatory variables (birding specialization and interest/activity. We further recommend using these measures in future research on species knowledge.

## Figures and Tables

**Table 1:** Sample characteristics split according to gender.

Demographic Variables	Gender	
Occupation	Male	Female
Student	337	682
Employee	348	525
Distance to next forest patch		
<1 km	393	620
1 to 5 km	276	532
6 to 10 km	18	55
>10 km	7	11
Garden owner		
yes	479	840
no	214	377
Hometown size		
<2000	133	184
2000–5000	132	271
5000–20,000	180	336
20,000–100,000	139	254
>100,000	108	172
Age		
Mean	33.46	31.41
SD	14.01	12.92

**Table 2:** Eigenvalues and explained variance (per factor and cumulative) of the explorative factor analysis.

Component	Eigen-Value	% Variance Explained	Cumulative Variance
1	3.347	55.78	55.78
2	0.861	14.36	70.13
3	0.661	11.02	81.15
4	0.424	7.07	88.22
5	0.415	6.91	95.13
6	0.292	4.87	100

**Table 3:** Results of the explorative factor analysis. Loadings of the respective items on the principal component 1 (PC1) are shown in descending order. We show the original German items and the English version.

Item Wording	PC1
I am interested in ornithology/science of birds. Ich interessiere mich für Vogelkunde.	0.846
How often do you read about birds? Wie oft lesen Sie über Vögel?	0.824
How often do you watch birds in nature? Wie oft beobachten Sie Vögel in der Natur?	0.800
The topic is important for me. Das Thema ist mir wichtig.	0.795
How often do you watch TV about birds? Wie oft schauen Sie sich Sendungen über Vögel an?	0.684
How often do you walk in nature? Wie oft gehen Sie in der Natur spazieren?	0.462

**Table 4:** Results of a General Linear Model with identification score as dependent variable and occupation, distance to next forest patch as fixed factors, and birding specialization and interest as covariates. Levene test: *p* = 0.163.

Source of Variance	df	Mean of Squares	F	*p*	Partial Eta^2^
Corrected model	6	6651.79	420.75	<0.001	0.576
Constant	1	4363.05	275.98	<0.001	0.129
Occupation	1	186.40	11.79	0.001	0.006
Distance to next forest patch	3	73.39	4.64	0.003	0.007
Interest/activity	1	2316.20	146.51	<0.001	0.073
Birding Specialization	1	13242.62	837.64	<0.001	0.310

Footnote: df = degrees of freedom; F = F statistics; *p* = significance; Partial Eta^2^ = measure of effect size.

## Data Availability

Data are available on the Open Science Framework under https://osf.io/u46w7/files/, accessed on 25 April 2021.

## Appendix A

**Table A1:** Bird species used in this study. The breeding pairs are only rough estimates based on the German breeding bird atlas [30].

English Name	Scientific Name	Order	Breeding Pairs
Great crested grebe	*Podiceps cristatus*	Podicipediformes	25,000
Grey heron	*Ardea cinerea*	Ciconiformes	30,000
White stork	*Ciconia ciconia*	Ciconiformes	4400
Common buzzard	*Buteo buteo*	Accipitriformes	100,000
Mallard	*Anas plathyrhynchos*	Anseriformes	200,000
Coot	*Fulica atra*	Gruiformes	90,000
Pheasant	*Phasianus colchicus*	Galliformes	250,000
Swift	*Apus apus*	Apodiformes	250,000
Lapwing	*Vanellus vanellus*	Charadriiformes	80,000
Black-headed gull	*Chroicocephalus ridibundus*	Charadriiformes	125,000
Cuckoo	*Cuculus canorus*	Cuculiformes	50,000
Tawny owl	*Strix aluco*	Strigiformes	55,000
Great spotted woodpecker	*Dendrocopos major*	Piciformes	800,000
Woodpigeon	*Columba palumbus*	Columbiformes	≈3 Mio
Jay	*Garrulus glandarius*	Passeriformes	500,000
Great tit	*Parus major*	Passeriformes	≈6 Mio
Robin	*Erithacus rubecula*	Passeriformes	≈4 Mio
Blackbird	*Turdus merula*	Passeriformes	≈8 Mio
Chaffinch	*Fringilla coelebs*	Passeriformes	≈8 Mio
Carrion crow	*Corvus corone*	Passeriformes	650,000
Magpie	*Pica pica*	Passeriformes	450,000
Nuthatch	*Sitta europaea*	Passeriformes	≈1.5 Mio
Starling	*Sturnus vulgaris*	Passeriformes	≈3.5 Mio
Blackcap	*Sylvia atricapilla*	Passeriformes	≈4 Mio
Chiffchaff	*Phylloscopus collybita*	Passeriformes	≈3 Mio
House sparrow	*Passer domesticus*	Passeriformes	≈4 Mio
Black redstart	*Phoenicurus ochruros*	Passeriformes	900,000
Yellowhammer	*Emberiza citrinella*	Passeriformes	≈1.5 Mio
